# Brain morphological changes in acquired hearing loss: A surface-based morphometry study

**DOI:** 10.1371/journal.pone.0343373

**Published:** 2026-03-25

**Authors:** Hye Ah Joo, Hwon Heo, Tae Uk Cheon, Yun Ji Lee, Yeonjoo Choi, Woo Hyun Shim, Hong Ju Park

**Affiliations:** 1 Department of Otorhinolaryngology-Head and Neck Surgery, Asan Medical Center, University of Ulsan College of Medicine, Seoul, Republic of Korea; 2 Department of Convergence Medicine, Asan Medical Center, University of Ulsan College of Medicine, Seoul, Republic of Korea; 3 Department of Otorhinolaryngology, Gangnam Severance Hospital, Yonsei University College of Medicine, Seoul, Republic of Korea; 4 Department of Otorhinolaryngology-Head and Neck Surgery, Soonchunhyang University Seoul Hospital, Seoul, Republic of Korea; 5 Department of Otorhinolaryngology-Head and Neck Surgery, Hallym University Kangnam Sacred Heart Hospital, Hallym University College of Medicine, Seoul, Republic of Korea; 6 Department of Radiology and Research Institute of Radiology, Asan Medical Center, University of Ulsan College of Medicine, Seoul, Republic of Korea; Universidad de Chile, CHILE

## Abstract

Prolonged auditory deprivation induces neuroplastic changes throughout the brain, including the auditory system. Understanding these structural alterations is crucial for optimizing auditory rehabilitation strategies. This study investigated brain morphological alterations associated with bilateral severe-to-profound sensorineural hearing loss (bilateral deafness; BD), focusing on cortical thickness (CT) and cortical volume (CV), and examined whether alterations in auditory-related cortical regions were associated with the duration of deafness (DoD) or hearing aid use (DoHA). High-resolution three-dimensional T1-weighted MRI data from 47 BD patients (≥10 years of hearing loss) and 73 normal hearing (NH) controls were retrospectively analyzed using surface-based morphometry (SBM) in FreeSurfer. Vertex-wise group comparisons of CT and CV were performed using a general linear model controlling for age. Partial correlation analyses were then conducted between CT/CV of eight auditory-related regions of interest and DoD/DoHA. Compared with the NH group, the BD group showed reduced CT in the bilateral superior temporal gyri and lateral occipital cortices, along with significant CV reductions in the bilateral superior temporal gyri, superior parietal cortices, and lateral occipital cortices. Notably, longer DoHA was positively correlated only with CT in the right superior temporal gyrus (r = 0.409, *p* = 0.005, FDR-adjusted *p* = 0.040). These findings demonstrate that long-term BD is associated with widespread cortical atrophy, affecting regions involved in auditory processing and the integration of somatosensory and visual information. Sustained hearing aid use may help preserve cortical structure, suggesting that timely auditory rehabilitation could slow neurodegeneration and potentially mitigate cognitive risks associated with hearing loss.

## Introduction

Neuroplasticity refers to adaptive brain modifications in response to a changing environment and the subsequent reconfiguration of functional connectivity [1]. Hearing loss (HL) is not merely a dysfunction of the auditory periphery but also causes functional and morphological changes throughout the entire auditory system, from the periphery to the cortex [2]. Damage to peripheral auditory input induces neuroplastic changes in downstream brain regions of the auditory pathway, including the cochlear nucleus, superior olivary complex, inferior colliculus, medial geniculate nucleus (thalamus), and auditory cortex [3]. An imbalance in cochlear output across frequencies, along with resulting changes in excitation and inhibition, disrupts the homeostatic processes of the central auditory system and ultimately leads to reorganization of cortical tonotopic areas [4,5].

Structural magnetic resonance imaging (MRI) studies following HL have provided insights, but the anatomical substrates of these changes in the human brain remain unclear, and a cause–effect relationship remain unclear. Several studies have shown that hearing impairment is associated with reduced gray matter (GM) volume in the primary auditory cortex among older individuals [6–8]. Longitudinal studies including older adults with hearing impairment have demonstrated accelerated rates of GM volume decline in the right temporal lobe and reduced prefrontal cortex GM volume and thickness, which correlated with poor speech perception in noise, as assessed using voxel-based morphometry (VBM) [9,10]. Whole brain atrophy with reduced white matter (WM) volume has been identified in older individuals with peripheral HL [11]. Diffuse tensor imaging studies have revealed reduced fractional anisotropy values, indicating more diffuse transmission, in several WM pathways leading to and from the auditory cortex, including the right superior and inferior longitudinal fasciculi, corticospinal tract, inferior fronto-occipital tract, superior occipital fasciculus, and anterior thalamic radiation [8,12,13]. Therefore, HL is associated with reduced GM volumes in the auditory processing areas of the superior temporal and prefrontal cortices, along with decreased WM tract integrity in the central auditory pathways.

The findings are less consistent for younger individuals. Neuschwander et al. [14] observed that higher hearing thresholds were associated with a thinner cortex in the left Heschl’s gyrus not only in older adults but also in younger and middle-aged adults, independent of age-related cognitive decline and age-dependent whole brain atrophy. Conversely, Boyen et al. [15] found that poorer hearing thresholds were associated with increased GM volume in the temporal and limbic lobes and decreased GM volume in the frontal lobe in middle-aged adults with peripheral HL. Other VBM-based studies have revealed preserved GM volume in the auditory cortex in early congenital deafness [16,17].

Despite the growing prevalence of HL across various age groups (even in early adulthood) [18] and its substantial social costs as a modifiable risk factor for dementia [19], research on structural neuroplasticity across various age groups and large study populations remains limited. Given that HL-induced changes in speech and auditory perception are reflected in neuroplasticity, understanding the structural brain modifications following HL is crucial. This knowledge could help elucidate the pathways through which peripheral sensory impairments contribute to brain changes and clarify whether appropriate auditory interventions can prevent or reverse these anatomical shifts.

Furthermore, auditory rehabilitation with hearing aids (HA) or cochlear implants (CI) has been associated with a reduced risk of long-term cognitive decline [20–22]. However, the impact of hearing restorative devices on structural brain changes and the factors influencing brain morphology following HL remain unclear. Shiell et al. [23] reported that longer durations of hearing aid use were associated with reduced cross-modal reorganization in deaf patients. Therefore, we hypothesized that a shorter duration of HL or longer HA use would mitigate or delay morphometric changes, particularly in the auditory-related cortical regions, among participants with HL, regardless of age.

This study aimed to investigate brain morphological changes associated with peripheral HL by comparing adult patients with acquired bilateral severe-to-profound sensorineural HL (bilateral deafness, BD) with controls with normal hearing (NH) in both ears. We primarily focused on changes in cortical thickness (CT) and cortical volume (CV), assessed using surface-based morphometry (SBM) with FreeSurfer. Furthermore, we examined whether alterations in the auditory-related cortical regions were associated with the duration of deafness (DoD) or the duration of hearing aid use (DoHA) within the BD group.

As supporting information, we also assessed subcortical GM volumes, including the hippocampus and amygdala, using FreeSurfer, given the well-established association between HL and dementia [19,24,25]. In addition, complementary VBM analyses were performed using the FMRIB Software Library (FSL) for cortical GM volume comparison, as VBM and SBM are known to complement each other, enhancing the accuracy of detecting cortical morphological changes [26].

## Materials and methods

This retrospective study compared patients (aged ≥18 years) with acquired bilateral severe-to-profound sensorineural HL to controls with NH in both ears. We reviewed data of patients who visited the outpatient clinic of the Department of Otorhinolaryngology-Head and Neck Surgery at Asan Medical Center, a tertiary referral center, who underwent hearing tests and brain MRI between November 2018 and June 2023. The control group consisted of patients who visited the clinic for other otologic complaints (without HL, tinnitus, or other hearing problems) and participants from a public medical data cohort provided by employees of our center.

BD was defined as symmetric, bilateral, severe-to-profound HL acquired after the development of language (post-lingual), characterized by mean air conduction pure-tone averages (mPTA, calculated as the average of hearing thresholds at 500, 1000, 2000, and 4000 Hz) ≥70 dB HL, with a DoD exceeding 10 years in both ears. NH was defined as mPTA < 25 dB HL in both ears. Among the selected participants, those with unsuitable brain MR images were excluded based on the following criteria: 1) absence of T1-weighted scans, 2) scans without any planes thinner than 1.5 mm, 3) scans that did not cover the whole brain, and 4) poor image quality that rendered registration impossible. Additionally, patients with a history of neurological or psychiatric disorders, intellectual disability, brain surgery, epilepsy, or psychotropic medication use were excluded.

This study was approved by the Institutional Review Board of Asan Medical Center (Project No. 2023−1106; Receipt Nos. S2023-1816–0001, −0002, −0003, −0005, and −0007). The requirement for informed consent was waived due to the low risk associated with retrospective data review and the use of anonymized images. Data were accessed for research purposes between September 1, 2023 and February 28, 2025. Identifiable participant information was accessible to the authors during the initial data collection phase; however, all data were subsequently anonymized to ensure confidentiality, and the authors had no access to personally identifiable information thereafter. All methods were performed in accordance with the relevant guidelines and regulations, including the Declaration of Helsinki.

### Study cohorts

This study included 47 patients with BD and 73 NH controls. Asymmetry in the pure-tone thresholds between both ears did not exceed 20 dB HL at one frequency, 15 dB HL at two frequencies, or 10 dB HL at three of the frequencies between 250 and 4000 Hz for any participants. Additionally, all participants had normal or corrected-to-normal vision. The DoD, defined as the period during which both ears were deaf, and the DoHA, defined as the period of HA were used in at least one ear, were retrospectively reviewed within the BD group.

### MRI acquisition

MRI scans were obtained using a 3T MRI scanner (Ingenia, Philips Medical Systems, Best, The Netherlands) with an eight-channel coil. High-resolution T1-weighted three-dimensional (3D) gradient echo images were acquired in axial, sagittal and coronal planes with the following parameters: TR/TE = 6.4–6.9/2.9–3.2 ms; FA = 9.0; FOV = 270; matrix = 256 × 256; thickness = 1–3 mm. To ensure consistent morphometric accuracy, only scans with a slice thickness less than 1.5 mm were included in the final analysis. The total scan time was approximately 30 min, and images were stored on a Picture Archiving and Communication System (PACS) for each patient. All scans were reviewed to check for motion artifacts and to rule out gross neuropathology.

### Image processing and measurements using FreeSurfer

**Fig 1:**
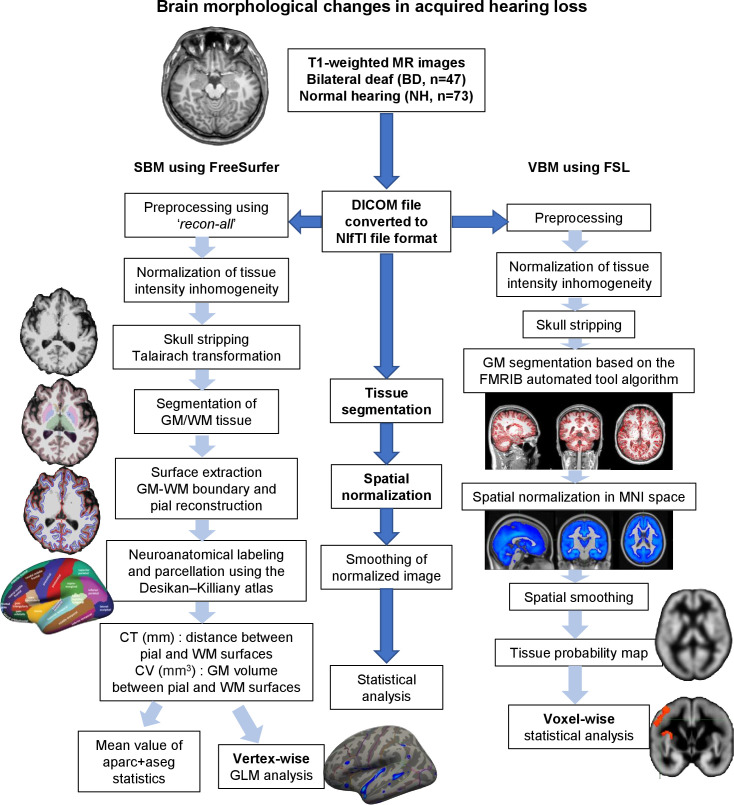
Overview of data processing steps used for the SBM and VBM analyses in this study. CSF, cerebrospinal fluid; CT, cortical thickness; CV, cortical volume; DICOM, Digital Imaging and Communications in Medicine; FSL, FMRIB software library; GLM, General Linear Model; GM, gray matter; MNI, Montreal Neurological Institute; MR, magnetic resonance; NIfTI, Neuroimaging Informatics Technology Initiative; SBM, surface-based morphometry; VBM, voxel-based morphometry; WM, white matter.

Brain cortical surface reconstruction and volumetric segmentation were performed using the open-source FreeSurfer image analysis suite (version 7.3.0, Athinoula A. Martinos Center for Biomedical Imaging, Harvard University, Boston, Massachusetts; http://surfer.nmr.mgh.harvard.edu/). FreeSurfer enables independent CT and CV assessments at the vertex level without human intervention, providing a more detailed and differentiated analysis of brain cortical anatomy than the conventional VBM approach [27,28]. A flowchart of the processing pipeline using FreeSurfer is shown in Fig 1.

T1-weighted MR images of the participants were downloaded from the PACS system, and the Digital Imaging and Communications in Medicine files were converted to the Neuroimaging Informatics Technology Initiative format. The images were then preprocessed using a standard processing stream in FreeSurfer called *recon-all*. The *recon-all* pipeline consists of 29 steps leading to cortical reconstruction and segmentation [27]. The preliminary steps include tissue intensity inhomogeneity normalization, skull stripping, Talairach transformation (2D spatial normalization from native space to standard spherical surface space), segmentation of GM/WM tissue and the assignment of neuroanatomical labels to each voxel using the Desikan–Killiany atlas [29], resulting in the parcellation of 34 distinct cortical regions of interest (ROIs) for each hemisphere. For each image, the accuracy of GM-WM boundary and pial reconstructions was visually inspected by the investigator (H.A.J.), blinded to the identities of the participants during preprocessing. The accuracy of cortical ribbon and tissue segmentation was evaluated using Freeview, a visualization tool included with FreeSurfer. The cortical ribbon was visually inspected and rated as “failed” if there was either overestimation or underestimation, using the T1 image as reference [30]. CT (in mm) was estimated as the distance between the pial and WM surfaces, and CV (in mm^3^) was calculated as the total volume of GM between the pial and WM surfaces.

Additionally, subcortical GM volumes were measured using FreeSurfer’s volume-based subcortical stream. Hippocampal and amygdalar volumes were further quantified using an automated module that segments their subfields [31,32].

### Statistical analysis

Demographic and clinical variables were compared between groups using independent t-tests and chi-square tests in SPSS (version 24.0, IBM Corp., Armonk, NY, USA), with statistical significance set at *p*-value < 0.05.

For SBM, the general linear model (GLM) in FreeSurfer was used to assess group differences in CT and CV, with age included as a covariate (GLM_g2v1) to control for age-related cortical atrophy. Vertex-wise group comparisons were conducted separately for each hemisphere, and false discovery rate (FDR) correction was applied to control for type I error due to multiple comparisons (*p* < 0.05).

Additionally, partial correlation analyses were conducted within the BD group to investigate whether CT/CV alterations in auditory-related cortical regions (perisylvian region) were associated with DoD or DoHA. Based on previous studies [14,33,34], eight auditory-related ROIs were defined on the Desikan–Killiany atlas, including the bilateral superior temporal gyri, banks of the superior temporal sulcus, transverse temporal gyri, and temporal poles, which are considered the regions most likely to be affected by HL-induced changes. The mean CT and CV values of each ROI were extracted directly from FreeSurfer’s automatic segmentation according to the parcellation atlas. Partial correlation analyses with DoD/DoHA were then performed while controlling for age, followed by subsequent FDR correction (*p* < 0.05).

Subcortical GM volumes were also extracted from FreeSurfer’s automatic segmentation and independent t-tests were used to compare mean subcortical GM volumes between groups.

### Additional VBM analysis

A complementary VBM analysis conducted using FSL software (version 6.0.7.14, FMRIB, Oxford, UK, https://fmrib.ox.ac.uk/fsl/) to compare cortical GM volume. SBM, which is spatially aligned based on the cortical folding pattern, provides a more sensitive and precise assessment of cortical morphology at the vertex level. In this study, VBM was performed only to provide complementary information on CV, offering sensitivity to distributed voxel-wise volumetric differences relative to the primary SBM results. Due to the well-known limitations of VBM in accurately analyzing subcortical structures, only cortical changes were considered significant and included in the analysis. The process includes preprocessing (skull stripping, signal nonuniformity correction) and key steps such as tissue segmentation, spatial normalization, image smoothing, and statistical analysis (Fig 1) [26]. Segmentation was performed using the FMRIB automated segmentation tool, which applies bias correction, skull stripping, and registration to prior tissue probability maps based on GM, WM, and cerebrospinal fluid [35].

Next, 3D spatial normalization of the segmented GM images was conducted, transforming them from native space to Montreal Neurological Institute space using linear and nonlinear image registration tools [36]. The investigator (W.H.S.), blinded to participant identities, conducted quality checks after each step. Following the spatial smoothing process with a Gaussian smoothing kernel (3 mm) to reduce interindividual variation and improve the repeatability of the measurements, probability density values for cortical GM tissue were obtained for each voxel in the segmented GM image [26]. Finally, voxel-wise statistical analyses were performed, comparing the probability density values for GM tissue between the groups while controlling for age. Multiple comparisons were corrected using Threshold-Free Cluster Enhancement with 5000 permutations [37].

## Results

The demographic and clinical characteristics of the study participants are summarized in Table 1. No significant differences in age or sex distribution were observed between the two groups. The BD group had a mean DoD of 28.55 years in both ears and a mean DoHA of 15.68 years. Regarding the etiologies of HL in the BD group, 19 patients (40.4%) had idiopathic HL related to aging or other unknown causes, followed by chronic otitis media (34.0%) and sudden HL (10.6%). No significant differences in total intracranial volume or mean CT in both hemispheres were found between the groups, as estimated by FreeSurfer. Within the BD group, no significant differences in total intracranial volume or mean CT were observed among subgroups classified according to HL etiology.

**Table 1:** Demographic and clinical characteristics.

	Bilateral deafness (BD)	Normal hearing (NH)	*p*-value
Participants (n)	47	73	
Age (year)	52.23 ± 16.96 (18–84)	50.19 ± 8.68 (24–72)	0.448
Sex (n)			0.916
Male	23 (48.9%)	35 (47.9%)	
Female	24 (51.1%)	38 (52.1%)	
Pure tone audiometry (Mean AC threshold, dB HL)			
Left ear	104.7 ± 12.8	13.8 ± 6.8	< 0.001
Right ear	103.9 ± 12.3	13.1 ± 6.8	< 0.001
Speech audiometry (Mean WRS, %)			
Left ear	6.9 ± 17.0	99.9 ± 0.9	< 0.001
Right ear	5.8 ± 13.6	99.8 ± 1.0	< 0.001
Duration of deafness (year)	28.55 ± 15.73 (10–70)		
Duration of HA use (year)	15.68 ± 11.79 (0–50)		
Hearing loss etiology (n)			
Idiopathic	19 (40.4%)		
Chronic otitis media	16 (34.0%)		
Sudden hearing loss	5 (10.6%)		
Genetic disorders	4 (8.5%)		
Head trauma	2 (4.3%)		
Noise exposure	1 (2.1%)		
FreeSurfer estimated values			
eTIV (mm^3^)	1503620.82 ± 200693.37	1492347.19 ± 192644.05	0.761
Mean cortical thickness (mm)			
Left hemisphere	2.42 ± 0.12	2.44 ± 0.07	0.206
Right hemisphere	2.41 ± 0.12	2.44 ± 0.07	0.196

AC, air conduction; eTIV, estimated total intracranial volume; HA, hearing aids; WRS, word recognition score.

### Group-based differences in CT

**Fig 2:**
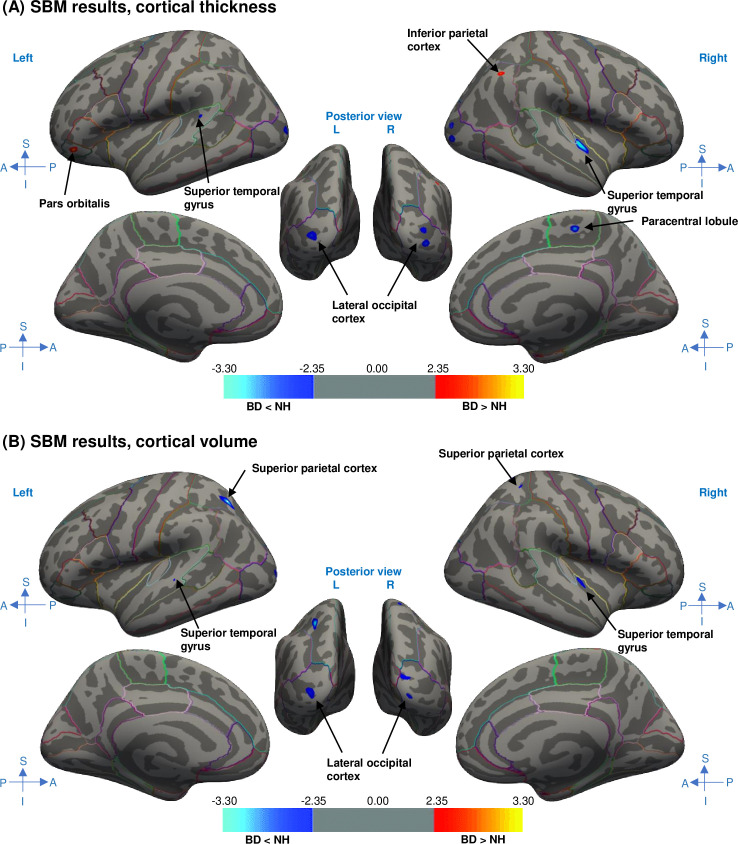
Vertex-wise group-based differences in cortical thickness (A) and volume (B) adjusted for age. Regions were delineated according to the Desikan–Killiany atlas. Dark gray represents sulci, and light gray represents gyri. Statistical significance is indicated by a color map, where the value corresponds to -log(*p*-value) (e.g., a value of 2 represents *p* = 0.01). FDR correction was subsequently applied (*p* < 0.05). BD, bilateral deafness; FDR, false discovery rate; NH, normal hearing; SBM, surface-based morphometry.

In the GLM group analysis of CT, including age as a covariate, the BD group exhibited significantly thinner cortices compared to the control group in several regions (Fig 2A). Specifically, the left superior temporal gyrus, left lateral occipital cortex, right superior temporal gyrus, right paracentral lobule, and right lateral occipital cortex were thinner in the BD group than in the control group; however, the left pars orbitalis and right inferior parietal cortex were significantly thicker in the BD group. The estimated mean CT for ROIs in each group, based on the Desikan–Killiany atlas is presented in S1 Table.

### Group-based differences in CV

In the GLM group analysis of CV, including age as a covariate, significant differences were identified between the groups in several regions (Fig 2B). The BD group exhibited significant CV reductions in the bilateral superior temporal gyri, superior parietal cortices, and lateral occipital cortices compared to the control group. The estimated mean CV for ROIs in each group, based on the Desikan–Killiany atlas is presented in S2 Table.

### Correlation between CT/CV of auditory-related ROIs and DoD/DoHA in the BD group

**Fig 3:**
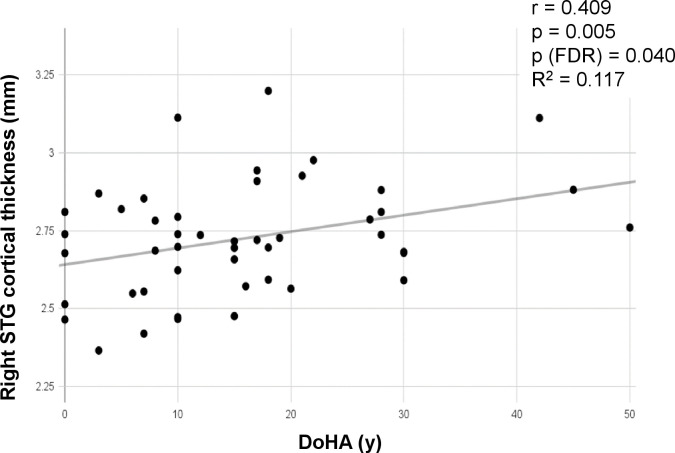
Correlation between CT of the right STG and DoHA. In partial correlation analyses conducted in the BD group, controlling for age, a statistically significant positive correlation was identified between the CT of the right STG and DoHA. BD, bilateral deafness; CT, cortical thickness; DoHA, duration of hearing aid use; STG, superior temporal gyrus.

Partial correlation analyses were conducted to assess whether the CT or CV of the predefined eight auditory-related ROIs were associated with DoD or DoHA in the BD group, adjusting for age (Table 2). Among these, only the CT of the right superior temporal gyrus showed a significantly positive correlation with DoHA in BD the group (r = 0.409, *p* = 0.005, FDR-adjusted *p* = 0.040) (Fig 3).

**Table 2:** Partial correlation of auditory-related cortical thickness and volume with duration of deafness and hearing aid use.

Bilateral deafness (n = 47)	Duration of deafness			Duration of hearing aids use		
Region of interest	r	*p*-value	*p*-value (FDR)	r	*p*-value	*p*-value (FDR)
**Cortical thickness**						
Left superior temporal	0.094	0.536	0.858	0.133	0.377	0.679
Left bankssts	0.113	0.453	0.858	0.100	0.509	0.679
Left transverse temporal	−0.030	0.845	0.946	0.026	0.865	0.893
Left temporal pole	−0.371	0.011*	0.088	−0.020	0.893	0.893
Right superior temporal	0.041	0.788	0.946	0.409	0.005*	0.040*
Right bankssts	0.156	0.301	0.803	0.300	0.043	0.172
Right transverse temporal	0.210	0.160	0.640	0.026	0.865	0.893
Right temporal pole	−0.010	0.946	0.946	0.190	0.205	0.547
**Cortical volume**						
Left superior temporal	0.117	0.437	0.576	0.117	0.437	0.576
Left bankssts	0.101	0.504	0.576	0.101	0.504	0.576
Left transverse temporal	0.159	0.292	0.576	0.159	0.292	0.576
Left temporal pole	−0.209	0.163	0.576	−0.209	0.163	0.576
Right superior temporal	0.054	0.724	0.724	0.054	0.724	0.724
Right bankssts	0.131	0.384	0.576	0.131	0.384	0.576
Right transverse temporal	0.190	0.205	0.576	0.190	0.205	0.576
Right temporal pole	−0.168	0.265	0.576	−0.168	0.265	0.576

bankssts, banks of the superior temporal sulcus; *p*-value (FDR), false discovery rate (FDR)-adjusted *p*-value; Partial correlation analyses were performed controlling for age and FDR correction was applied.

* indicates a *p*-value < 0.05.

### Group-based differences in subcortical GM volumes

Compared with the control group, the BD group exhibited significantly smaller GM volumes in the bilateral thalamus (*p* < 0.001 in the left and *p* = 0.003 in the right), hippocampus (*p* = 0.041 in the left and *p* = 0.029 in the right), amygdala (*p* = 0.020 in the left and *p* = 0.007 in the right), and in the right pallidum (*p* = 0.026) (Table 3). The most pronounced volume reduction in the left hippocampus was observed in the hippocampal body and tail, whereas in the right hippocampus, it was primarily seen in the hippocampal body.

**Table 3:** Mean volumes of subcortical gray matter structures and group comparison.

Subcortical volume (mm^3^)	Bilateral deafness (BD)	Normal hearing (NH)	*p*-value
Left thalamus	7045.99 ± 1011.21	7760.06 ± 994.51	< 0.001*
Right thalamus	6750.10 ± 936.28	7244.16 ± 807.55	0.003*
Left caudate	3208.32 ± 502.26	3293.71 ± 406.96	0.309
Right caudate	3383.45 ± 502.91	3401.58 ± 406.43	0.829
Left putamen	4431.38 ± 718.60	4669.46 ± 515.51	0.053
Right putamen	4601.06 ± 722.23	4771.84 ± 511.45	0.163
Left pallidum	1938.84 ± 282.77	1990.08 ± 219.90	0.268
Right pallidum	1926.06 ± 291.76	2042.60 ± 265.97	0.026*
Left hippocampus	3436.20 ± 482.26	3601.27 ± 386.59	0.041*
Left hippocampal head	1714.73 ± 280.73	1769.43 ± 210.55	0.256
Left hippocampal body	1183.70 ± 158.91	1243.11 ± 135.86	0.031*
Left hippocampal tail	537.77 ± 86.73	588.73 ± 82.84	0.002*
Right hippocampus	3547.27 ± 451.55	3717.20 ± 381.20	0.029*
Right hippocampal head	1767.93 ± 253.22	1855.00 ± 225.54	0.052
Right hippocampal body	1207.03 ± 141.68	1261.89 ± 132.03	0.033*
Right hippocampal tail	572.31 ± 78.59	600.31 ± 78.02	0.058
Left amygdala	1645.57 ± 230.61	1734.02 ± 179.94	0.020*
Right amygdala	1732.31 ± 250.49	1848.11 ± 205.60	0.007*
Left accumbens area	416.14 ± 115.85	437.54 ± 101.02	0.287
Right accumbens area	483.73 ± 105.31	515.95 ± 83.41	0.080

Data are presented as mean ± standard deviation. *P*-value was calculated using the independent t-test.

* indicates a *p*-value < 0.05.

### Additional VBM analysis of cortical GM volumes

In additional VBM analyses comparing voxel-wise cortical GM volumes between the BD and NH groups, while controlling for age, the findings partially corroborated the results from SBM and revealed several additional regions of difference. The BD group exhibited significantly reduced GM volume compared to NH controls, primarily in the left middle and inferior frontal gyri, left somatosensory cortex (inferior portion, including precentral and postcentral gyri), bilateral superior and middle temporal gyri, and occipital cortices (*p* < 0.001, S1 Fig). In contrast, the BD group showed a significant increase in GM volume in the upper portion of the left precentral gyrus (*p* < 0.01, S2 Fig).

## Discussion

With the rising prevalence of acquired HL, understanding brain changes through synaptic modifications and neural circuit rewiring is crucial. To our knowledge, this is the largest study analyzing brain structural changes using SBM in adult patients with post-lingual BD, encompassing 47 individuals, aged 18–84 years. We primarily observed reduced CT in the bilateral superior temporal gyri and lateral occipital cortices, along with significant CV reductions in the bilateral superior temporal gyri, superior parietal cortices, and lateral occipital cortices in the BD group than in the control group. These regions are primarily associated with auditory processing and the integration of somatosensory and visual information. Findings regarding CV partially aligned with the complementary VBM analysis results, further supporting the structural alterations identified through SBM. Additionally, we observed smaller subcortical volumes in the thalamus, hippocampus, and amygdala in the BD group than in the control group, as assessed using FreeSurfer. Finally, in the BD group, a significant positive correlation was found between DoHA and the CT of the right superior temporal gyrus among the predefined eight auditory-related ROIs.

One of our key findings was the positive correlation between DoHA and the CT of the right superior temporal gyrus in the BD group, controlling for age. This suggests that sustained auditory input via HAs may help delay auditory cortical atrophy, even in prolonged severe-to-profound HL. This observation supports the notion of experience-dependent neuroplasticity, whereby continued peripheral auditory stimulation helps preserve cortical integrity. Furthermore, it underscores the potential clinical importance of early and consistent HA use in mitigating cortical degeneration associated with auditory deprivation.

A critical public health question is whether HL-induced brain morphological changes can be reversed through intervention. Interestingly, several studies have reported evidence of cortical plasticity following auditory rehabilitation in individuals with deafness. Pereira-Jorge et al. [20] demonstrated increased CT in multimodal integration regions, particularly at the caudal end of the superior temporal sulcus, angular gyrus, and inferior parietal gyrus/superior temporal gyrus/insula, after a year of continuous HA use in patients with BD. Positron emission tomography studies have also revealed increased activation in the auditory cortex in CI users, particularly during speech processing [38,39]. Our recent work demonstrated a significant increase in CV in the contralateral superior temporal gyrus after CI, with this volumetric increase positively correlating with post-CI speech perception [40]. Collectively, these findings indicate that effective auditory rehabilitation may not only delay but also partially reverse brain structural changes associated with HL. The BD group exhibited significantly reduced CT and smaller CV in the bilateral superior temporal gyri, consistent with VBM findings. The superior temporal gyrus plays a key role in auditory processing, including speech and language functions [41]. It is essential for extracting meaningful linguistic features from speech input and maintaining short-term auditory sensory memory [42]. The prolonged absence of auditory sensory input in BD likely resulted in significant shrinkage of the superior temporal gyrus, reflecting a potential loss of neurons and/or glial cells due to reduced neural stimulation. This aligns with previous studies reporting lower GM density and glucose metabolism in the primary auditory cortex of postlingually deaf individuals [43,44].

The parietal cortex, located between auditory, visual, and somatosensory regions, is crucial for multisensory and sensorimotor integration, including spatial navigation, motor planning, spatial attention, egocentric and allocentric reference frame use, working memory, and action execution [45]. In our study, the BD group showed cortical thickening in the right inferior parietal cortex, consistent with its role in cross-modal plasticity and increased activity following sensory loss [45]. In contrast, CV was significantly reduced in the bilateral superior parietal cortices, which support spatial processing, movement planning, and sign language processing in individuals with HL [46]. We hypothesize that prolonged auditory deprivation may decrease information flow to these regions due to decreased social interaction, thereby hindering use-dependent structural maintenance.

Previous studies suggest that HL induces visual-auditory cross-modal reorganization, increasing resting-state occipital cortex activity to compensate for auditory deprivation [43,47,48]. Colonization of auditory areas by visual speech processing and increased neural responsiveness to visual stimuli has also been observed. However, in our cohort, CT and CV were significantly reduced in the bilateral lateral occipital cortices, consistent with VBM findings. Similar reductions of GM in the occipital lobe have been reported in hearing-impaired individuals in previous studies [15,49]. Given the prolonged auditory deprivation in our cohort (mean DoD: 28.55 years), this decline may reflect a gradual reduction of GM volume in the auditory cortex over time, which visual cortex plasticity alone cannot fully compensate [50]. Strong auditory-visual connectivity exists, with visually modulated neurons frequently observed outside the deep cortical layers of the primary auditory cortex [51]. Additionally, deafness has been associated with impaired visual functions, including higher visual temporal thresholds and poorer visual discrimination [52–54]. These findings support the “deficiency theory,” which suggests that the loss of one sensory modality can disrupt the development of another [52]. Furthermore, occipital cortex activity has been positively correlated with auditory speech recovery following CI in adults with deafness, highlighting the importance of visuoauditory synergy for cross-modal plasticity [47].

Our VBM analyses corroborated the SBM findings, showing similar patterns of cortical GM volume decline, with additional cortical differences in the left frontal and somatosensory areas. Specifically, reduced GM volume was observed in the left middle and inferior frontal gyri. The middle frontal gyrus is crucial for reorienting attention and memory retrieval, functions that may be impaired in patients with BD due to decreased speech perception [55]. The left inferior frontal gyrus, which contains Broca’s area, is associated with the motor aspects of speech and expressive language production [56]. Prolonged HL may diminish input to Broca’s area due to difficulties in oral communication and reduced social interaction. This lack of stimulation may hinder use-dependent retention and contribute to atrophy in this region. A significant GM volume reduction was also observed in the inferior portion of the left precentral and postcentral gyri, whereas an increase was noted in the upper portion of the left precentral gyrus. Similarly, Sun et al. [57] reported lower GM probabilities in the bilateral thalami, superior, middle, and inferior temporal cortices, as well as in central cortical regions linked to the sensation and movement of the lips, tongue, and larynx in individuals with post-lingual deafness compared to the controls. Decreased auditory input adversely affects speech production, leading to declines in articulatory skills and difficulties in acquiring new vocabulary. Our VBM analysis revealed GM volume reductions corresponding to regions of the homunculus associated with the lips, tongue, pharynx, and larynx, potentially reflecting these declines [58]. Conversely, increased GM volume in the upper portion of the left precentral gyrus in patients with BD corresponds to the homunculus for the hand and arm, suggesting enhanced use of their hands for communication.

Moreover, cognitive impairment arises from disrupted connections among the hippocampus, amygdala, and prefrontal cortex, which are crucial for memory and emotional learning [59]. HL has been linked to reduced hippocampal volume and accelerated decline, with an inverse relationship observed between hearing thresholds and hippocampal volume, even in cognitively normal individuals [60–62]. Hippocampal atrophy is a marker of neurodegeneration and can precede the clinical onset of Alzheimer’s disease, the most common cause of dementia [60]. Similarly, amygdala volume reduction has been associated with synaptic dysfunction and disease progression [63,64], with its magnitude comparable to hippocampal atrophy and related to global illness severity. Our findings suggest that prolonged deafness may contribute to volume reduction in these subcortical structures, potentially increasing dementia risk [19].

Recent evidence indicates that cochlear amplifier dysfunction may be an important mechanism linking HL to brain atrophy within the extended network involved in effortful hearing [65]. Loss of outer hair cells has been associated with accelerated cell death in the central structures, including the cingulate cortex, hippocampus, and amygdala, thereby contributing to cognitive decline [66].

This study has several limitations. First, its retrospective design may have introduced biases in data collection and interpretation. Second, cognitive function was not objectively assessed when analyzing cortical and subcortical changes. Future research should incorporate standardized cognitive evaluation tools to better assess HL-related risks. Third, covariates that may contribute to microangiopathy and consequently to GM alterations, such as lifestyle factors or chronic underlying conditions, were not fully assessed, limiting subgroup analyses. Fourth, the lack of standardized anatomical definitions for functional brain regions across brain atlases posed challenges. Future studies with larger, prospective cohorts with varying HL severities and longitudinal designs are required to better assess neuroplastic changes over time. Standardized MRI protocols will ensure data consistency. Additionally, central hearing abilities, speech-in-noise tasks, and higher cognitive functions should be evaluated to provide more comprehensive insights. Finally, randomized controlled trials are necessary to examine the effects of HAs and CIs on cortical morphology, cognition, and quality of life, guiding evidence-based public health strategies.

## Conclusions

SBM analysis revealed significant cortical changes associated with structural degeneration in patients with BD. Additionally, these patients exhibited reduced subcortical volumes in the thalamus, hippocampus, and amygdala. Notably, a longer DoHA was significantly correlated with increased CT in the right superior temporal gyrus in individuals with BD, suggesting a delay in brain morphological changes within the auditory cortex. These findings highlight the importance of adequate auditory rehabilitation for individuals with peripheral HL, as such interventions may help mitigate the risk of cognitive decline associated with hearing impairment.

## Supporting information

S1 Fig Statistical parametric maps from whole-brain VBM analysis (BD < NH). The analysis was conducted after adjusting for age, revealing regions with cortical GM volume decline in the BD group compared to the control group. The statistical significance threshold was set at *p* < 0.001 with TFCE correction for multiple comparisons. The BD group showed reduced GM volume primarily in the left middle frontal gyrus and inferior frontal gyrus (A), as well as in the left somatosensory cortex, including the precentral and postcentral gyri (B). Bilateral GM volume reductions were also observed in the superior temporal gyrus, middle temporal gyrus (C), and occipital cortex (D). (A: anterior, P: posterior, S: superior, I: inferior, L: left, R: right). BD, bilateral deafness; GM, gray matter; NH, normal hearing; TFCE, Threshold-Free Cluster Enhancement; VBM, voxel-based morphometry.
(TIFF)

S2 Fig Statistical parametric maps from whole-brain VBM analysis (BD > NH). The analysis was conducted after adjusting for age effects, revealing regions with cortical GM volume increase in the BD group compared to the control group. The statistical significance threshold was set at *p* < 0.001 with Threshold-Free Cluster Enhancement correction for multiple comparisons. The BD group showed increased GM volume in the upper portion of the left precentral gyrus. BD, bilateral deafness; GM, gray matter; NH, normal hearing; TFCE, Threshold-Free Cluster Enhancement; VBM, voxel-based morphometry.
(TIFF)

S1 Table Mean values of cortical thickness in each region of interest and group comparison. (DOCX)

S2 Table Mean values of cortical volume in each region of interest and group comparison. (DOCX)
