# Supplementary figures and images for: Brain morphological changes in acquired hearing loss: A surface-based morphometry study
Source: PLoS One. 2026 Mar 25;21(3):e0343373. doi: 10.1371/journal.pone.0343373 (PMC13016313; doi:10.1371/journal.pone.0343373)

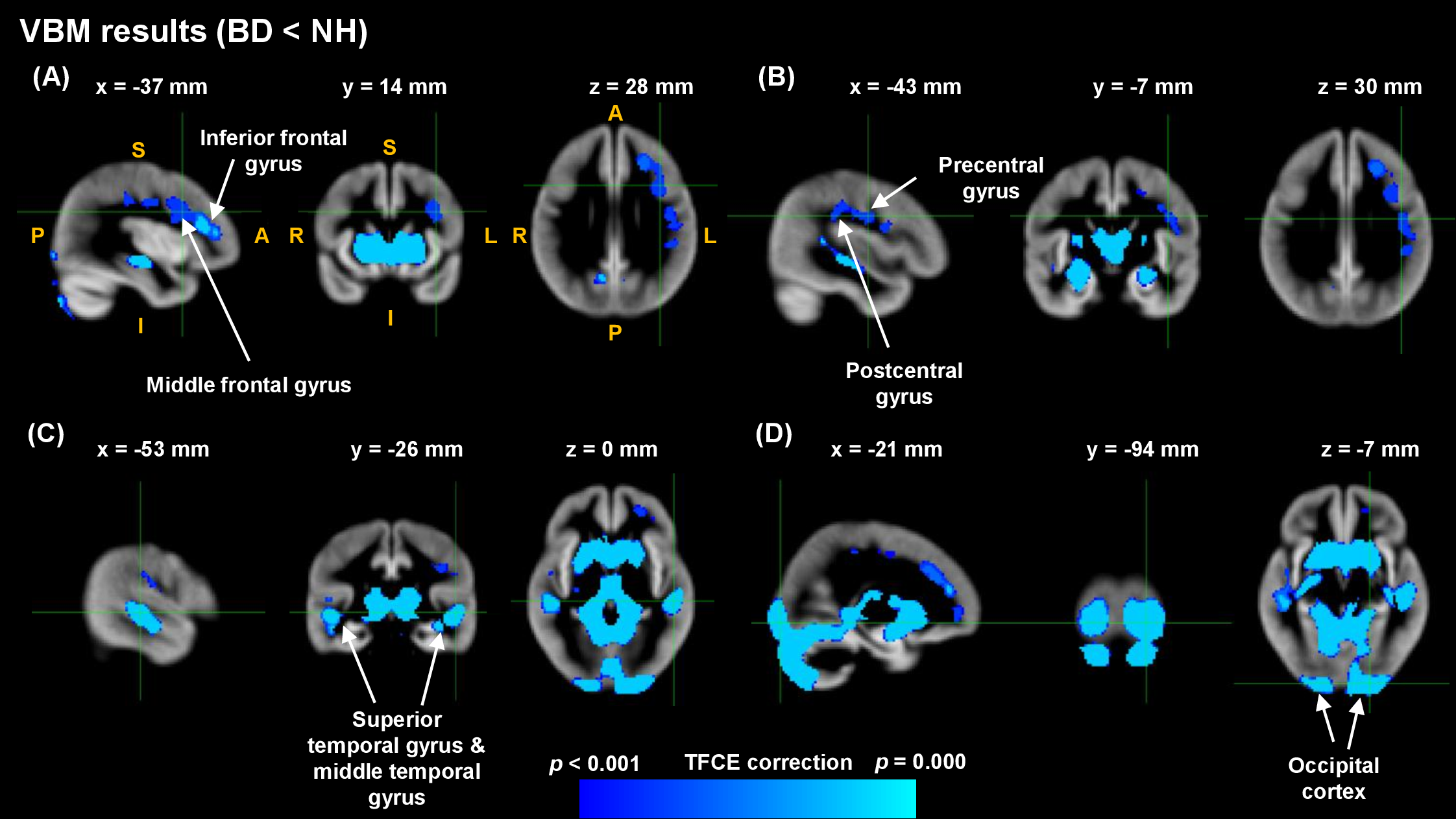

Supplement: S1 Fig — The analysis was conducted after adjusting for age, revealing regions with cortical GM volume decline in the BD group compared to the control group. The statistical significance threshold was set at p < 0.001 with TFCE correction for multiple comparisons. The BD group showed reduced GM volume primarily in the left middle frontal gyrus and inferior frontal gyrus (A), as well as in the left somatosensory cortex, including the precentral and postcentral gyri (B). Bilateral GM volume reductions were also observed in the superior temporal gyrus, middle temporal gyrus (C), and occipital cortex (D). (A: anterior, P: posterior, S: superior, I: inferior, L: left, R: right). BD, bilateral deafness; GM, gray matter; NH, normal hearing; TFCE, Threshold-Free Cluster Enhancement; VBM, voxel-based morphometry. (TIFF) [file pone.0343373.s001.tiff]

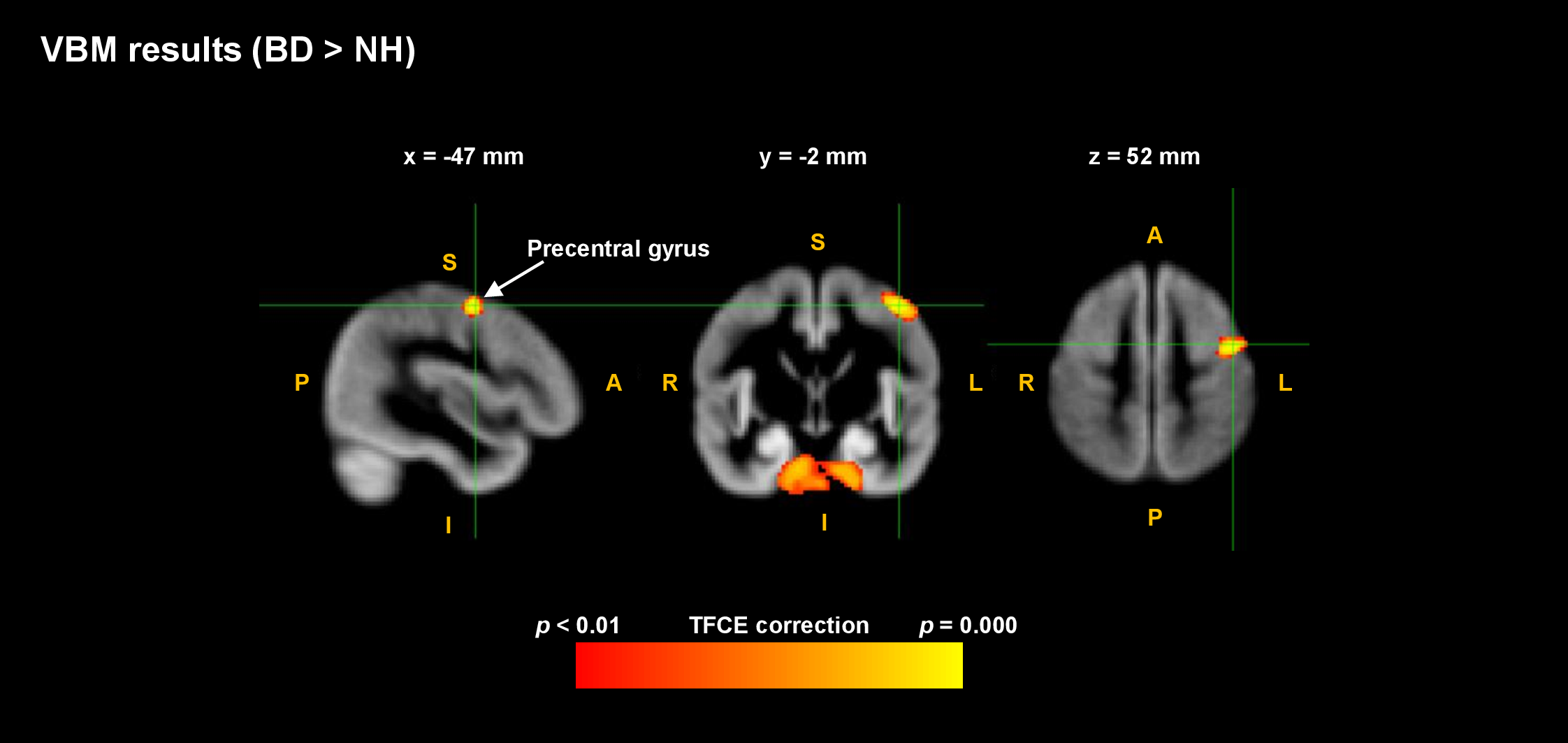

Supplement: S2 Fig — The analysis was conducted after adjusting for age effects, revealing regions with cortical GM volume increase in the BD group compared to the control group. The statistical significance threshold was set at p < 0.001 with Threshold-Free Cluster Enhancement correction for multiple comparisons. The BD group showed increased GM volume in the upper portion of the left precentral gyrus. BD, bilateral deafness; GM, gray matter; NH, normal hearing; TFCE, Threshold-Free Cluster Enhancement; VBM, voxel-based morphometry. (TIFF) [file pone.0343373.s002.tiff]
