# Supplementary material for: Brain morphological changes in acquired hearing loss: A surface-based morphometry study
Source: PLoS One. 2026 Mar 25;21(3):e0343373. doi: 10.1371/journal.pone.0343373 (PMC13016313; doi:10.1371/journal.pone.0343373)
Supplement: S1 Table — (DOCX) [file pone.0343373.s003.docx]

**S1 Table.** **Mean values of cortical thickness in each region of interest and group comparison.**

|  | **Left hemisphere** | | | | | **Right hemisphere** | | | |  |
| --- | --- | --- | --- | --- | --- | --- | --- | --- | --- | --- |
|  |  | | Thickness (mm), mean ± SD | | |  | Thickness (mm), mean ± SD | | |  |
|  | Regions | BD | | NH | *p*-value | Regions | BD | NH | *p*-value |  |
|  | Transverse temporal cortex | 2.15±0.27 | | 2.32±0.22 | 0.000 | Precentral gyrus | 2.46±0.17 | 2.57±0.12 | 0.000 |  |
|  | Precentral gyrus | 2.50±0.18 | | 2.60±0.12 | 0.001 | Superior temporal gyrus | 2.72±0.18 | 2.84±0.14 | 0.000 |  |
|  | Lateral occipital cortex | 2.05±0.14 | | 2.11±0.10 | 0.004 | Transverse temporal cortex | 2.19±0.27 | 2.38±0.19 | 0.000 |  |
|  | Superior temporal gyrus | 2.68±0.16 | | 2.76±0.12 | 0.006 | Lateral occipital cortex | 2.09±0.15 | 2.18±0.12 | 0.001 |  |
|  | Rostral anterior cingulate cortex | 2.73±0.18 | | 2.66±0.17 | 0.028 | Rostral anterior cingulate cortex | 2.85±0.25 | 2.72±0.19 | 0.003 |  |
|  | Inferior parietal cortex | 2.36±0.17 | | 2.41±0.11 | 0.077 | Superior parietal cortex | 2.06±0.17 | 2.12±0.10 | 0.031 |  |
|  | Lingual gyrus | 1.86±0.14 | | 1.82±0.11 | 0.078 | Caudal anterior-cingulate cortex | 2.49±0.22 | 2.41±0.21 | 0.034 |  |
|  | Postcentral gyrus | 1.96±0.13 | | 2.00±0.10 | 0.089 | Paracentral lobule | 2.42±0.19 | 2.49±0.14 | 0.043 |  |
|  | Caudal middle frontal gyrus | 2.44±0.16 | | 2.48±0.13 | 0.091 | Postcentral gyrus | 1.95±0.13 | 2.00±0.11 | 0.043 |  |
|  | Superior parietal cortex | 2.11±0.16 | | 2.15±0.10 | 0.148 | Medial orbital frontal cortex | 2.48±0.13 | 2.44±0.11 | 0.064 |  |
|  | Banks superior temporal sulcus | 2.47±0.22 | | 2.52±0.16 | 0.156 | Caudal middle frontal gyrus | 2.42±0.15 | 2.46±0.12 | 0.079 |  |
|  | Paracentral lobule | 2.44±0.21 | | 2.49±0.15 | 0.157 | Isthmus-cingulate cortex | 2.29±0.18 | 2.24±0.15 | 0.108 |  |
|  | Medial orbital frontal cortex | 2.42±0.14 | | 2.39±0.10 | 0.195 | Inferior parietal cortex | 2.33±0.16 | 2.37±0.11 | 0.125 |  |
|  | Caudal anterior-cingulate cortex | 2.51±0.24 | | 2.46±0.19 | 0.222 | Temporal pole | 3.77±0.29 | 3.84±0.23 | 0.127 |  |
|  | Posterior-cingulate cortex | 2.47±0.18 | | 2.43±0.13 | 0.232 | Pericalcarine cortex | 1.49±0.19 | 1.45±0.12 | 0.140 |  |
|  | Isthmus-cingulate cortex | 2.30±0.20 | | 2.27±0.13 | 0.286 | Frontal pole | 2.77±0.34 | 2.69±0.19 | 0.142 |  |
|  | Lateral orbital frontal cortex | 2.67±0.13 | | 2.64±0.10 | 0.310 | Lingual gyrus | 1.90±0.15 | 1.87±0.10 | 0.159 |  |
|  | Precuneus cortex | 2.32±0.18 | | 2.35±0.12 | 0.324 | Banks superior temporal sulcus | 2.52±0.20 | 2.57±0.14 | 0.174 |  |
|  | Superior frontal gyrus | 2.70±0.18 | | 2.72±0.12 | 0.380 | Entorhinal cortex | 3.51±0.29 | 3.56±0.27 | 0.332 |  |
|  | Pars orbitalis | 2.69±0.22 | | 2.66±0.18 | 0.389 | Middle temporal gyrus | 2.84±0.18 | 2.87±0.11 | 0.383 |  |
|  | Pars triangularis | 2.43±0.18 | | 2.45±0.11 | 0.438 | Supramarginal gyrus | 2.40±0.16 | 2.42±0.09 | 0.433 |  |
|  | Supramarginal gyrus | 2.43±0.15 | | 2.45±0.11 | 0.503 | Precuneus cortex | 2.30±0.20 | 2.32±0.11 | 0.474 |  |
|  | Parahippocampal gyrus | 2.70±0.29 | | 2.67±0.26 | 0.511 | Cuneus cortex | 1.80±0.17 | 1.78±0.11 | 0.493 |  |
|  | Pars opercularis | 2.52±0.19 | | 2.54±0.12 | 0.520 | Inferior temporal gyrus | 2.82±0.17 | 2.84±0.12 | 0.527 |  |
|  | Rostral middle frontal gyrus | 2.31±0.15 | | 2.30±0.09 | 0.574 | Pars triangularis | 2.43±0.17 | 2.44±0.10 | 0.568 |  |
|  | Temporal pole | 3.78±0.25 | | 3.76±0.22 | 0.628 | Posterior-cingulate cortex | 2.43±0.16 | 2.42±0.14 | 0.678 |  |
|  | Middle temporal gyrus | 2.81±0.16 | | 2.82±0.10 | 0.770 | Insula | 3.01±0.19 | 3.00±0.17 | 0.684 |  |
|  | Insula | 2.98±0.20 | | 2.97±0.17 | 0.801 | Rostral middle frontal gyrus | 2.32±0.13 | 2.31±0.10 | 0.729 |  |
|  | Pericalcarine cortex | 1.43±0.17 | | 1.43±0.11 | 0.818 | Fusiform gyrus | 2.78±0.15 | 2.77±0.11 | 0.769 |  |
|  | Entorhinal cortex | 3.49±0.28 | | 3.48±0.24 | 0.865 | Pars opercularis | 2.56±0.17 | 2.55±0.13 | 0.769 |  |
|  | Inferior temporal gyrus | 2.83±0.15 | | 2.83±0.11 | 0.893 | Lateral orbital frontal cortex | 2.62±0.14 | 2.62±0.10 | 0.772 |  |
|  | Frontal pole | 2.72±0.22 | | 2.73±0.21 | 0.914 | Superior frontal gyrus | 2.73±0.19 | 2.74±0.11 | 0.818 |  |
|  | Cuneus cortex | 1.76±0.17 | | 1.75±0.11 | 0.965 | Parahippocampal gyrus | 2.62±0.25 | 2.62±0.20 | 0.959 |  |
|  | Fusiform gyrus | 2.76±0.16 | | 2.76±0.12 | 0.988 | Pars orbitalis | 2.65±0.22 | 2.65±0.16 | 0.967 |  |

BD, bilateral deafness; NH, normal hearing; SD, standard deviation.

The *p*-value was calculated using the independent t-test.

Each cortical region of interest is segmented according to the Desikan–Killiany atlas.
