# Supplementary material for: Brain morphological changes in acquired hearing loss: A surface-based morphometry study
Source: PLoS One. 2026 Mar 25;21(3):e0343373. doi: 10.1371/journal.pone.0343373 (PMC13016313; doi:10.1371/journal.pone.0343373)
Supplement: S2 Table — (DOCX) [file pone.0343373.s004.docx]

**S2 Table.** **Mean values of cortical volume in each region of interest and group comparison.**

|  | **Left hemisphere** | | | | | **Right hemisphere** | | | |  |
| --- | --- | --- | --- | --- | --- | --- | --- | --- | --- | --- |
|  |  | | Volume (mm^3^), mean ± SD | | |  | Volume (mm^3^), mean ± SD | | |  |
|  | Regions | BD | | NH | *p*-value | Regions | BD | NH | *p*-value |  |
|  | Precentral gyrus | 12200.83±1885.02 | | 13305.47±1605.80 | 0.001 | Transverse temporal cortex | 742.04±153.11 | 888.96±147.27 | 0.000 |  |
|  | Transverse temporal cortex | 995.94±238.90 | | 1138.51±234.50 | 0.002 | Lateral occipital cortex | 10769.45±1997.71 | 11907.05±1780.83 | 0.001 |  |
|  | Inferior parietal cortex | 11364.04±2166.38 | | 12377.00±1648.17 | 0.004 | Precentral gyrus | 11932.11±1850.73 | 13007.66±1476.55 | 0.001 |  |
|  | Superior temporal gyrus | 11286.57±1917.15 | | 12178.18±1731.94 | 0.009 | Superior temporal gyrus | 10530.72±1700.18 | 11501.95±1396.29 | 0.001 |  |
|  | Supramarginal gyrus | 9980.23±1585.22 | | 10792.48±1911.61 | 0.017 | Postcentral gyrus | 8150.09±1280.81 | 8774.15±1213.20 | 0.008 |  |
|  | Caudal middle frontal gyrus | 5168.66±1043.76 | | 5664.53±1233.86 | 0.024 | Banks superior temporal sulcus | 1932.68±441.36 | 2120.53±368.56 | 0.013 |  |
|  | Lateral occipital cortex | 10825.19±2000.55 | | 11491.86±1576.50 | 0.044 | Inferior parietal cortex | 13164.04±2701.00 | 14270.40±2130.89 | 0.014 |  |
|  | Paracentral lobule | 3313.34±509.83 | | 3497.00±476.48 | 0.047 | Posterior-cingulate cortex | 2927.04±512.60 | 3156.21±536.57 | 0.021 |  |
|  | Pars opercularis | 3965.70±742.32 | | 4235.48±723.02 | 0.051 | Inferior temporal gyrus | 10241.64±1588.03 | 10861.70±1887.29 | 0.064 |  |
|  | Banks superior temporal sulcus | 2195.43±522.90 | | 2370.08±443.60 | 0.052 | Caudal middle frontal gyrus | 4976.72±1000.32 | 5325.77±1011.88 | 0.066 |  |
|  | Fusiform gyrus | 9115.98±1437.16 | | 9581.38±1395.41 | 0.076 | Precuneus cortex | 9098.64±1616.50 | 9610.52±1258.89 | 0.069 |  |
|  | Postcentral gyrus | 8524.77±1339.41 | | 8936.15±315.33 | 0.093 | Parahippocampal gyrus | 1794.66±274.58 | 1876.95±246.20 | 0.090 |  |
|  | Superior frontal gyrus | 19883.74±3010.74 | | 20773.48±2669.87 | 0.093 | Middle temporal gyrus | 11135.49±2091.68 | 11728.19±1690.14 | 0.091 |  |
|  | Isthmus-cingulate cortex | 2325.87±442.31 | | 2449.10±358.97 | 0.097 | Frontal pole | 1153.49±182.13 | 1104.70±145.16 | 0.107 |  |
|  | Middle temporal gyrus | 10228.85±2105.75 | | 10806.47±1392.74 | 0.101 | Fusiform gyrus | 9111.64±1474.06 | 9530.71±1386.32 | 0.118 |  |
|  | Pars triangularis | 3314.53±702.79 | | 3497.41±550.74 | 0.114 | Paracentral lobule | 3588.98±627.17 | 3749.74±496.18 | 0.121 |  |
|  | Rostral middle frontal gyrus | 13403.45±2281.00 | | 13989.89±1882.69 | 0.128 | Superior frontal gyrus | 18954.13±2971.78 | 19715.51±2554.01 | 0.138 |  |
|  | Posterior-cingulate cortex | 2933.26±578.54 | | 3071.71±455.34 | 0.147 | Superior parietal cortex | 11544.06±2028.35 | 12010.19±1558.76 | 0.159 |  |
|  | Cuneus cortex | 2723.13±708.76 | | 2875.70±433.19 | 0.190 | Rostral middle frontal gyrus | 13947.19±2615.66 | 14552.86±2115.17 | 0.166 |  |
|  | Lateral orbital frontal cortex | 6938.38±1052.00 | | 7151.18±719.34 | 0.191 | Insula | 6535.91±947.34 | 6753.21±804.87 | 0.181 |  |
|  | Inferior temporal gyrus | 11003.17±2054.99 | | 11473.26±1832.78 | 0.194 | Supramarginal gyrus | 9016.68±1772.78 | 9361.70±1248.76 | 0.249 |  |
|  | Precuneus cortex | 8941.34±1524.66 | | 9243.33±1248.72 | 0.238 | Lateral orbital frontal cortex | 6927.87±1139.10 | 7106.90±776.29 | 0.348 |  |
|  | Insula | 6800.77±1000.08 | | 6997.00±812.85 | 0.241 | Cuneus cortex | 3101.60±670.11 | 3204.12±536.38 | 0.356 |  |
|  | Superior parietal cortex | 12132.77±2019.46 | | 12509.37±1762.00 | 0.283 | Pars opercularis | 3474.36±629.99 | 3561.71±592.01 | 0.443 |  |
|  | Rostral anterior cingulate cortex | 2354.23±479.36 | | 2457.52±548.51 | 0.293 | Rostral anterior cingulate cortex | 1827.36±465.06 | 1889.59±488.76 | 0.466 |  |
|  | Entorhinal cortex | 1861.28±295.72 | | 1917.38±391.41 | 0.403 | Entorhinal cortex | 1745.34±301.93 | 1781.68±371.51 | 0.575 |  |
|  | Temporal pole | 2606.17±370.47 | | 2566.36±400.54 | 0.585 | Isthmus-cingulate cortex | 2303.40±466.90 | 2341.22±361.46 | 0.619 |  |
|  | Frontal pole | 939.00±178.48 | | 925.67±116.33 | 0.621 | Pars triangularis | 3983.87±837.75 | 4047.84±649.20 | 0.640 |  |
|  | Medial orbital frontal cortex | 4866.21±681.31 | | 4916.12±678.38 | 0.695 | Pars orbitalis | 2596.91±501.13 | 2558.66±390.92 | 0.641 |  |
|  | Lingual gyrus | 5674.83±1279.29 | | 5745.60±915.90 | 0.743 | Temporal pole | 2591.06±371.42 | 2622.79±361.32 | 0.643 |  |
|  | Pars orbitalis | 2155.32±403.19 | | 2172.11±278.08 | 0.803 | Lingual gyrus | 6270.64±1426.98 | 6375.70±1010.59 | 0.662 |  |
|  | Pericalcarine cortex | 1716.49±534.00 | | 1735.64±315.33 | 0.825 | Caudal anterior-cingulate cortex | 1900.13±392.01 | 1920.32±510.85 | 0.808 |  |
|  | Parahippocampal gyrus | 1969.64±282.69 | | 1974.12±274.44 | 0.931 | Pericalcarine cortex | 2070.85±528.57 | 2075.56±408.67 | 0.956 |  |
|  | Caudal anterior-cingulate cortex | 1533.06±449.42 | | 1537.22±445.39 | 0.960 | Medial orbital frontal cortex | 5414.74±695.62 | 5421.37±621.16 | 0.957 |  |

BD, bilateral deafness; NH, normal hearing; SD, standard deviation.

The *p*-value was calculated using the independent t-test.

Each cortical region of interest is segmented according to the Desikan–Killiany atlas.
